# The Buffalo Medical College—Session 1880-81

**Published:** 1880-10

**Authors:** 


### THE BUFFALO MEDICAL COLLEGE—SESSION OF 1880–81.

THE introductory lecture, which formally opens the regular course of medical lectures, will be delivered by Prof. E. V. Stoddard, on Wednesday evening, October 6th, at the Medical College. The lectures in the course will commence the following day. The curriculum will be enlarged the coming session, by the addition of a course of lectures on mental disorders, by Dr. Judson B. Andrews, the new Superintendent of the Buffalo State Asylum for the Insane. This important feature in medical education was urged several years ago, upon the State Medical Society by Prof. White, who will now be able to witness the successful introduction of instruction in this and allied subjects into the curriculum of the College over which he has so long and ably presided. We are also permitted to state that our colleague, Dr. Lucien Howe, has been invited to give a course of lectures on Ophthalmology, a selection which secures for the class superior advantages in this department.

The advantages offered by the Buffalo Medical College are well known and appreciated by the profession, and we join in congratulating the faculty upon its increasing reputation.

We hope the day is not far distant when we will witness the adoption of a graded course of instruction, such as Harvard has successfully inaugurated. In matters of medical education the standard is determined by the profession at large, and medical colleges in the standard of their requirements and the thoroughness of their instruction are but the exponent of that sentiment.

As journalists we will be prompt to recognize and endorse efforts here and elsewhere, tending to this important end. We wish the College deserved success in its labors for the advancement of medical science during the coming session.

